# DISC1 mouse models as a tool to decipher gene-environment interactions in psychiatric disorders

**DOI:** 10.3389/fnbeh.2013.00113

**Published:** 2013-09-03

**Authors:** Tyler Cash-Padgett, Hanna Jaaro-Peled

**Affiliations:** Department of Psychiatry and Behavioral Sciences, Johns Hopkins UniversityBaltimore, MD, USA

**Keywords:** DISC1, mouse models, gene-environment, schizophrenia, depression, immune activation, social stress

## Abstract

*DISC1* was discovered in a Scottish pedigree in which a chromosomal translocation that breaks this gene segregates with psychiatric disorders, mainly depression and schizophrenia. Linkage and association studies in diverse populations support *DISC1* as a susceptibility gene to a variety of neuropsychiatric disorders. Many *Disc1* mouse models have been generated to study its neuronal functions. These mouse models display variable phenotypes, some of them relevant to schizophrenia, others to depression. The *Disc1* mouse models are popular genetic models for studying gene-environment interactions in schizophrenia. Five different *Disc1* models have been combined with environmental factors. The environmental stressors employed can be classified as either early immune activation or later social paradigms. These studies cover major time points along the neurodevelopmental trajectory: prenatal, early postnatal, adolescence, and adulthood. Various combinations of molecular, anatomical and behavioral methods have been used to assess the outcomes. Additionally, three of the studies sought to rescue the resulting abnormalities. Here we provide background on the environmental paradigms used, summarize the results of these studies combining *Disc1* mouse models with environmental stressors and discuss what we can learn and how to proceed. A major question is how the genetic and environmental factors determine which psychiatric disorder will be clinically manifested. To address this we can take advantage of the many *Disc1* models available and expose them to the same environmental stressor. The complementary experiment would be to expose the same model to different environmental stressors. *DISC1* is an ideal gene for this approach, since in the Scottish pedigree the same chromosomal translocation results in different psychiatric conditions.

## Introduction

Both schizophrenia and depression have a strong genetic component but are also heavily influenced by the environment. Based on epidemiological studies, environmental schizophrenia risk factors include infections (especially during fetal development), obstetric complications (Mittal et al., 2008), advanced paternal age (Malaspina, 2001) adolescent cannabis use (Henquet et al., 2008), and several forms of stress (reviewed in Brown, 2011). In contrast to the wide variety of factors implicated in schizophrenia, stress in particular stands out as the major environmental risk factor for major depression. Early life stress, involving childhood maltreatment or traumatic events, can be particularly devastating (reviewed in Mann and Currier, 2006). Highly stressful life events, such as death of a family member or divorce are causally associated with onset of episodes of major depression (Kendler et al., 1999).

Here we review a group of studies that utilize mouse models of *DISC1*, a susceptibility gene for psychiatric disorders, to examine the interaction between genetic and environmental risk factors. We start by summarizing human data on the environmental factors most relevant to these studies, infections, and social stress, and their respective experimental paradigms in mice.

## Environment

### Environment: infections (reviewed in Brown, 2011)

Prenatal and early postnatal infections have been implicated in a number of major neurodevelopmental disorders. Direct infection of the fetus can cause serious congenital brain anomalies and mental retardation. Although schizophrenia typically arises in adolescence or young adulthood, it is increasingly regarded as neurodevelopmental in origin (Weinberger, 1996; Cannon et al., 2003; Allardyce et al., 2005; Jaaro-Peled et al., 2009; Shoji et al., 2012). This leads to the hypothesis that subtle perturbations in the developing brain, such as infection of the pregnant mother, may increase the risk of schizophrenia later on in the life of the offspring. Birth cohort studies have established an association between specific prenatal infections and elevated risk for schizophrenia. The largest effects have been attributed to influenza (Brown et al., 2004a), elevated maternal immunoglobulin G (IgG) to the parasite Toxoplasma gondii (Remington, 2006), and periconceptional genital or reproductive infection (Babulas et al., 2006).

A parsimonious explanation of how different viruses/parasites/bacteria increase the risk of the same disorder is suggested by the convergence of their pathogenic mechanism: stimulation of the maternal innate immune response and of cytokines in particular (Gilmore and Jarskog, 1997). Birth cohort studies support a role for excess maternal cytokines in the development of schizophrenia among offspring (Brown et al., 2004b). As with many proteins originally identified in the immune system, cytokines are also expressed in the nervous system and modulate many aspects of neural development and physiology (Szelenyi, 2001). Transplacental transfer of maternally produced cytokines and production of cytokines at the maternal-fetal interface both lead to an elevation of these molecules in the fetal brain (Meyer et al., 2009). Earlier inflammation is expected to have a more severe effect on brain development due to disruption of fundamental neurodevelopmental processes such as cell proliferation and differentiation, and may predispose the developing nervous system to fail in subsequent cell migration, target selection, and synapse maturation (Figure 6 in Meyer et al., 2007).

Although cytokines are the main suspects in mediating the detrimental effect of the pathogenic interaction between the maternal immune system and fetal brain, other mechanisms do exist. For example, while influenza is not believed to cross the placental barrier, it elicits IgG antibodies which do and subsequently cross-react with fetal brain antigens via molecular mimicry (Wright et al., 1999). Pathogens may also dysregulate brain development and function through a direct interaction between pathogen nucleic acids/proteins and (fetal) brain nucleic acids/proteins or modification of the fetal epigenome (Waterland and Michels, 2007). Some schizophrenia susceptibility genes may affect pathogen virulence, while the pathogens themselves may affect host genes and neural processes (Carter, 2009). For example, DISC1 regulates the microtubule network (Kamiya et al., 2005) that is exploited by viruses for intracellular trafficking.

Although both human and animal studies have mainly focused on maternal/prenatal immune activation, there is also evidence for postnatal infections increasing the risk of schizophrenia. Population-based studies have concluded that childhood viral infections of the central nervous system increase the risk of adult psychotic illness (Dalman et al., 2008; Khandaker et al., 2012).

### Environment: social stress

Starting as early as prenatal development, environmental stress dysregulates the homeostatic physiological stress response by modulating the hypothalamic-pituitary-adrenal (HPA) axis. This system normally responds to stress by producing glucocorticoids, which can bind receptors throughout the brain and act as transcription factors (Lupien et al., 2009). Neurons in the hypothalamus release corticotrophin releasing hormone (CRH) in response to a stressful environment. CRH triggers secretion of adrenocorticotropic hormone (ACTH) from the pituitary gland, in turn leading to production of glucocorticoids (cortisol in humans, corticosterones in mice) by the adrenal gland. Once the stressful situation has passed, negative feedback loops shut down this response and return the HPA axis to a set homeostatic point (O'connor et al., 2000).

A highly stressful social environment can cause maladaptive brain development and function from the prenatal period all the way through old age, and as a result chronic stress is a critical factor in the epidemiology of major mental illness. van Os et al. suggested that “psychotic syndromes can be understood as disorders of adaptation to social context” (Van Os et al., 2010). For example, schizophrenia risk is increased for people raised in an urban environment (Pedersen and Mortensen, 2006). Sub-optimal social conditions have been identified as important mediating factors, in particular low social capital and high social fragmentation (Allardyce et al., 2005; March et al., 2008). Immigrants also suffer from higher risk of psychosis (reviewed in Cantor-Graae and Selten, 2005); two potential explanations of this phenomenon are immigrants' experiences of discrimination and exposure to social defeat, both chronically stressful conditions. Childhood trauma may also increase the risk for psychosis, but more research is needed to establish stronger correlation (Morgan and Fisher, 2007).

The negative effects of chronic environmental stress manifest first in prenatal development, since maternal glucocorticoids circulate freely through the fetal brain. Significantly elevated maternal glucocorticoid levels can result in increased basal HPA axis activity in the offspring up to at least 10 years of age (O'connor et al., 2005). This heightened homeostatic set point results in increased risk for neurological and behavioral disturbances in the offspring (Stott, 1973). Since the human brain undergoes extended postnatal development, the effects of prenatal stress are moderated by the quality of the offspring's environment in early life.

Early life stress, often as a result of childhood maltreatment or traumatic events, decreases mental health in a dose-response fashion (Edwards et al., 2003). It causes dysregulation of the aforementioned stress response systems, leading to increased sensitivity to stress throughout life (reviewed in Heim and Binder, 2012), and has been associated with increased risk for mood and anxiety disorders, posttraumatic stress disorder, unipolar depression, and schizophrenia (Heim et al., 2010). Physiologically, early-life disruption of the HPA axis manifests as an array of abnormalities such as glucocorticoid resistance (Charmandari et al., 2004), increased CRH activity (Cratty et al., 1995), and increased levels of inflammation (McEwen, 1998). Human imaging studies have revealed further effects of early life stress on brain anatomy and function (Papagni et al., 2011). Animal models of early life stress such as variable schedules of maternal separation have provided direct evidence for the causal link between early adverse experience and later brain and behavior abnormalities. Maternal separation has been shown to increase glucocorticoid levels in separated mouse pups and to permanently change glucocorticoid receptor gene expression resulting in excessive glucocorticoid release under stress in adulthood (Anisman et al., 1998).

Detrimental effects on brain and behavior induced by adult chronic stress are reversed after a few weeks of no stress, in contrast to the permanent effects of early life stress (Avital and Richter-Levin, 2005). Exposure to an environmental event during development results in a stable phenotypic alteration in adulthood. This developmental “programming” can alter gene transcription via transcription factors or epigenetic mechanisms (Weaver et al., 2004). In mice, more attentive maternal care of the offspring modulates the selective methylation/demethylation of specific CpG dinucleotides of the glucocorticoid receptor gene (Oberlander et al., 2008). The resulting permanent decrease in glucocorticoid receptor density, selective to the hippocampus and prefrontal cortex, can increase HPA axis sensitivity to glucocorticoid-mediated negative feedback and lead to a reduction in plasma glucocorticoid levels and stress reactivity throughout the offspring's life (Weaver et al., 2004).

### Stress in adolescence (reviewed in Andersen and Teicher, 2008; Lupien et al., 2009)

Both animal and human studies show that adolescence is associated with higher basal and stress-induced activity of the HPA axis compared to both childhood (a stress hypo-responsive period) and adulthood (Vazquez and Akil, 1993; Gunnar et al., 2009). Adolescence is characterized by extensive changes in brain physiology (Andersen, 2003; Jaaro-Peled et al., 2009; Giedd and Rapoport, 2010; O'donnell, 2010), especially among regions involved in social function (Blakemore, 2008). Social stressors during this critical period can therefore potentially have significant long-term consequences on the “social brain” (Van Os et al., 2010). Notably, the brain areas undergoing development and hence most sensitive to stress during adolescence differ between rodents and humans. Rodent hippocampus continues to develop into adulthood, but in humans it is fully developed by 2 years of age (Giedd et al., 1996). While the frontal cortex and amygdala continue to develop in both, the process is more extensive in primates than in other species (Andersen, 2003). Adolescence is the peak age of onset for many psychiatric disorders, including anxiety, mood disorders, eating disorders, psychosis, and substance abuse (Paus et al., 2008).

## Gene × environment (G × E, reviewed in Duncan and Keller, 2011)

Gene × environment (G × E) implies synergistic interplay between genes and environment in causing a particular phenotype, where the effect of one is conditional on the other. Genes may modulate sensitivity to the environment, exemplified by a landmark study which demonstrated that a specific serotonin transporter polymorphism moderates sensitivity to life stress contributing to the pathogenesis of depression (Caspi et al., 2003). The environment can impact DNA sequences themselves as well as epigenetic control of risk genes.

Human G × E studies of early life stress and depression have focused mostly on a number of logical candidate genes involved in the serotonin system, neurotrophins, and most of interest to this review, the HPA axis (reviewed in Nugent et al., 2011). Polymorphisms in the glucocorticoid receptor and its regulator FK506 binding protein 5 (FKBP5) as well as in the CRH type I receptor interact with early life stress in predicting depression (Bradley et al., 2008) or in the case of FKBP5, post-traumatic stress disorder (Binder et al., 2008).

First-generation epidemiological G × E studies of psychotic psychiatric disorders have used indirect measures of genetic risk, such as blood relation to a person with schizophrenia, thus representing the complete genetic load including gene × gene interactions. Environmental variables examined in G × E studies include migration, urbanicity, obstetric complications, cannabis use, and stress (Van Os et al., 2008). It is difficult to design human studies that include enough participants to be able to test genetic and environmental risk factors as independent variables alongside any putative gene-environment interaction; any such synergistic interaction might be masked by other genetic and environmental factors. Thus, animal models are utilized in order to rigorously examine the mechanisms and outcomes of G × E interactions.

## Animal models

Epidemiological studies detect correlations which point to potential mechanisms and public health interventions, but causality can only be determined in animal models. These enable the controlled combination of a specific genetic risk factor (on an otherwise uniform genetic background) with a specific environmental risk factor, at a specific developmental time, and detailed observation of the emerging phenotypes over time. Of course, these models have clear limitations of their own; for example, when modeling brain diseases in animals, it is critical to take into account interspecies differences in the timing of brain development. A further complication is that the time course of development differs between different brain regions (cortex, limbic, etc.) and constituents (neurons, glia, blood-brain-barrier, etc.). Most relevant to this review, a large part of gestation in the mouse corresponds to the first half of human pregnancy, and many of the brain maturation events which happen during the second half of human gestation occur postnatally in mice (Clancy et al., 2007).

### Modeling infections in rodents

The effects of specific pathogens have been modeled in animals by prenatal infection with the influenza virus (Shi et al., 2003, 2005) and by adult infection with the Toxoplasma parasite (Vyas et al., 2007). More general effects of immune system activation have been modeled by injecting the bacterial endotoxin lipopolysaccharide (Miyazaki et al., 2004; Nogai et al., 2005) or proinflammatory cytokines (Nawa and Yamada, 2012). A strong innate immune response similar to that caused by viral infection can be induced by injection of a synthetic double-stranded RNA, polyriboinosinic-polyribocytidylic acid (polyI:C) (reviewed in Meyer and Feldon, 2012). Binding of polyI:C to Toll-like receptor 3 stimulates production of many pro-inflammatory cytokines and type I interferons (Okahira et al., 2005; Kumar et al., 2006). Maternal exposure to polyI:C alters cytokine levels in the three compartments of the maternal-fetal interface: placenta, amniotic fluid, and the fetus itself. PolyI:C has been widely used, as it has several advantages over live viruses; it is comparatively safe, and the intensity and timing of the immune response can be tightly controlled (Meyer and Feldon, 2012). However, polyI:C does not induce the full spectrum of immune responses induced by a virus. Prenatal polyI:C has been shown to induce distinct neuropathological and behavioral phenotypes depending on the gestational duration of exposure. For example, Meyer and Feldon hypothesize that the abnormalities induced by immune activation in early/middle pregnancy (embryonic day 9 in mice) are associated mainly with positive symptoms of schizophrenia, whereas the abnormalities caused by immune activation in late pregnancy (embryonic day 17 in mice) are more relevant to negative symptoms of schizophrenia (Bitanihirwe et al., 2010).

### Modeling social stress in rodents

As described above, social stress is particularly implicated in the pathogenesis of several major mental diseases. Maternal separation can be a devastating stressor, leading to an array of adult social and cognitive dysfunction (Schmidt et al., 2011). Mild chronic stress, entailed by serial exposure to mild but unpredictable stressors, is also a widely used model for depression (Hill et al., 2012). Social stress as applied to rodents can be subdivided to prenatal, early postnatal (until weaning), adolescent or adult.

#### Social isolation

Agonistic confrontations are not the only mediator of social stress in mammals. Positive social interactions are critical to healthy mental development. In humans, separation and social withdrawal are risk factors for psychiatric disorders (Allardyce et al., 2005). Both maternal separation and long-term social isolation in mice produce a phenotype characterized by cognitive and social-affective dysfunction, such as impaired prepulse inhibition (PPI) and novel object recognition as well as increased anxiety and aggressive behavior (Fone and Porkess, 2008; Niwa et al., 2011).

#### Social defeat

Social defeat paradigms involve agonistic interaction between two animals. Two mice (usually male) are put into a cage with a removable separator. Upon removal of the separator the two mice fight one another, and a clear “victor” is established in most cases. The defeated animal experiences social stress, and repeated exposures to this stressor have been linked to a robust social-affective dysfunction phenotype similar to depression, characterized by anhedonia, helplessness, hyperactivity, and social-avoidance (Kudryavtseva et al., 1991; Venzala et al., 2012). Exposure to social defeat can be one-time, intermittent, or chronic.

### Gene × environment × timing

The timing of an environmental factor (whether immune activation or stress) is critical and must be taken into account in experimental design and data interpretation. Dynamic, rapid neurodevelopmental processes occur during the pre- and early post-natal periods and to some extent also in adolescence. Each brain region is maximally sensitive to a given environmental factor during critical periods of high plasticity, which differ between species. Analogous to the typical adolescent or adult onset of psychiatric symptoms, behavioral effects of early-life interventions in experimental animals may become evident only in adulthood.

### Gene × environment studies using DISC1 mouse models

G × E studies in psychiatry are a relatively new field of research, as discovery of risk genes has only started in this millennium and the mouse models based on the genetic findings are not yet fully established. One of the most extensively characterized risk genes for psychiatric disorders is *DISC1*. *DISC1* was discovered in a Scottish pedigree in which a chromosomal translocation that breaks this gene segregates with psychiatric disorders (Millar et al., 2000). Although the acronym *DISC1* means *Disrupted in schizophrenia-1*, members of the pedigree were diagnosed as suffering from a variety of psychiatric disorders: recurrent major depression, schizophrenia, bipolar disorder and adolescent conduct disorder with a combined LOD score of 7.1 (Blackwood et al., 2001). Subsequent association and linkage studies in a variety of populations have confirmed *DISC1* as a susceptibility gene not only to those adult-onset disorders but also to autism (Chubb et al., 2008). DISC1 is a hub protein which regulates many aspects of development and function of the nervous system and as such mutations in it contribute to various brain disorders (Chubb et al., 2008; Brandon and Sawa, 2011; Porteous et al., 2011). The type of DISC1 mutation, mutations in other genes, and/or environmental factors may determine the specific symptoms.

#### DISC1 mouse models

One of the tools to study the biology of *DISC1* and its relevance to psychiatric disorders is mouse models in which this gene has been manipulated. Numerous *DISC1* mouse models have been generated and characterized for histological, anatomical, neurochemical, and behavioral phenotypes (for review, see Jaaro-Peled, 2009; Brandon and Sawa, 2011; Johnstone et al., 2011). Each model can be considered significantly unique from the others considering the genetic manipulation, the assays used to characterize it, and the particular lab environment; this diversity significantly complicates comparison between models. Theoretically similar assays may yield different results for different *DISC1* models or even for the same model under different lab conditions. Still, each of these genetic models without any environmental intervention show some abnormalities relevant for schizophrenia and/or mood disorders such as: parvalbumin and synaptic deficits, enlarged lateral ventricles, disturbed dopaminergic system, cognitive and emotional behavioral deficits. When looking for a suitable genetic model for G × E studies, it may be useful select G and E conditions which do not *individually* produce impaired phenotypes in the hope of G × E interaction producing a novel, emergent phenotype. There is a substantial pool of *Disc1* mouse models that could be potentially useful for such studies.

So far the *DISC1* G × E studies have been done with either *Disc1* point mutants (Clapcote et al., 2007) or with transgenic mice expressing C'-truncated (as in the original Scottish pedigree) dominant negative (DN) human DISC1 (on top of the endogenous wild-type mouse Disc1) under either the alpha calmodulin kinase II (CaMK) promoter or the prion protein promoter (PrP). The CaMK promoter induces expression of the DN-DISC1 in the pyramidal neurons of the forebrain (Tsien et al., 1996), whereas the PrP promoter induces its expression in most brain regions and cell types (Asante et al., 2002). Presumably due to *DISC1* being one of the most established risk genes for psychiatric disorders, its clear involvement in brain development, and the availability of many mouse models, more G × E studies have been performed using DISC1 mouse models than with any other risk gene. Here we will review five studies, starting with the studies using DN-DISC1 mouse models (Table 1) and then moving to the studies using *Disc1* point mutants (Table 2).

**Table 1:** **Summary of G × E in DN-DISC1 mouse models**.

	**Age:**	**Embryonic**			**Neonatal**				**Adolescent**			
		**Abazyan et al., 2010**			**Ibi et al., 2010; Nagai et al., 2011**				**Niwa et al., 2013**			
	**Condition:**	**G**	**E**	**G × E**	**G**	**E**	**G × E**	**G × E drug rescue**	**G**	**E**	**G × E**	**G × E drug rescue**
	**Manipulation:**	**DN-DISC1**	**E9 PolyI:C (5 mg/kg i.p.)**		**DN-DISC1**	**neonatal PolyI:C**		**Antipsychotics**	**DN-DISC1**	**Social isolation**		**GR antagonist**
Locomotion	OFT							Haloperidol, clozapine	(METH) =	(METH) =	Y	Normalized
Cognition and Memory	PPI	=	=	N	=	=	N		=	=	Y	Normalized
	Y maze	=	=	N	=	=	Y					
	NORT	=	=	N	=	=	Y	Clozapine				
Depression and Anxiety	FST	=	=	Y					=	=	Y	Normalized
	Sociability	=	=	Y								
	EPM	=	=	Y								
	OFT	=	=	Y								
Anatomy	LV Volume	↑	↑	Y (smaller increase)					=			
	Spine density	=	=	N								
Histology	Nissl stain								= (FC)	= (FC)	N	
	BrdU				=	=	Y (GCL)					
	Parvalbumin				=	=	Y (mPFC)					
Neurochemistry	Cytokine secretion	↑ IL-1B, IL-5	↑ IL-1B, IL-4, IL-5	Y (IL-2 only)								
	Corticosterone (stress response)	No recovery	No recovery	Y (smaller increase)					=	=	Y	
	Dopaminergic	=	=	=					=	=	Y (FC, not NAc)	Normalized

The down arrow (↓) indicates decrease, the up arrow (↑) indicates increase or augmentation compared to wild-type controls without environmental risk factor; Y indicates a significant G × E interaction (gene-environment interactions exacerbated behavioral deficits unless otherwise specified), N indicates the absence of a significant G × E interaction; G is the gene manipulated condition; E is the environment manipulated condition; G × E is the gene and environment manipulated condition. BrdU, bromodeoxyuridine; EPM, elevated plus maze; FC, frontal cortex; FST, forced swim test; GCL, granule cell layer of the hippocampus; GR, glucocorticoid receptor; IL, interleukin; i.p., intraperitoneal; LV, lateral ventricle; METH, methamphetamine; mPFC, medial prefrontal cortex; NAc, nucleus accumbens; NORT, novel object recognition test; OFT, open field test; PPI, prepulse inhibition.

**Table 2:** **Summary of G × E in point mutant *Disc1* mouse models**.

	**Age:**	**Embryonic**							**Adult**					
		**Lipina et al., 2013**							**Haque et al., 2012**					
	**Condition:**	**G**	**E**	**G × E**	**G**	**E**	**G × E**	**G × E drug rescue**	**G**	**E**	**G × E**	**G**	**E**	**G × E**
	**Manipulation:**	**Q31L**	**E9 PolyI:C (5 mg/kg i.v.)**		**L100P**	**E9 PolyI:C (2.5 mg/kg i.v.)**		**Anti-IL-6**	**Q31L**	**Chronic social defeat**		**L100P**	**Chronic social defeat**	
Locomotion	OFT	=	↑	N	=	=	N		=	=	N	=	=	Y
Cognition and memory	PPI	↓	↓	N	=	=	Y	Normalized	=	=	N	↓	=	N
	LI	=	↓	N	↓	↓	Y	Normalized	↓	=	N	↓	=	N
	NORT	=	=	N	=	=	Y							
Depression and anxiety	FST	=	=	N					=	↑	N	=	↑	N
	SC								↓	↓	N	↓	↓	N
	Sociability				=	=	Y		↓	↓	N	=	↓	N
	EPM (% closed arm)								↑	↑	N	↑	↑	Y
Neurochemistry	Cytokine secretion		↑ IL-6	N		↑ IL-6	Y							

The down arrow (↓) indicates decrease, the up arrow (↑) indicates increase or augmentation compared to wild-type controls without environmental risk factor; Y indicates a significant G × E interaction (gene-environment interactions exacerbated behavioral deficits), N indicates the absence of a significant G × E interaction; G is the gene manipulated condition; E is the environment manipulated condition; G × E is the gene and environment manipulated condition. EPM, elevated plus maze; FST, forced swim test; IL, interleukin; i.v., intravenous; LI, latent inhibition; NORT, novel object recognition test; OFT, open field test; PPI, prepulse inhibition; SC, sucrose consumption.

#### Inducible CaMK-DN-DISC1 × prenatal polyI:C (Abazyan et al., 2010)

The CaMK-DN-DISC1 genetic model displays brain and behavioral abnormalities including enlarged lateral ventricles, decreased protein levels of endogenous mouse DISC1, decreased levels of cortical dopamine and fewer parvalbumin-positive neurons in the cortex (Pletnikov et al., 2008; Ayhan et al., 2011). Behavioral abnormalities differ between males and females: males show both spontaneous and psychostimulant-induced hyperactivity in the open field as well as altered social interaction, whereas females exhibit impaired spatial memory.

PolyI:C (5 mg/kg) was injected to pregnant dams at embryonic day 9 (E9), modeling the complex scenario of infection affecting the maternal-fetal interface. This timing of polyI:C injection, corresponding to middle/end of first trimester of human pregnancy, has been widely used (Meyer et al., 2006). Abazyan et al. performed intraperitoneal injections—in contrast to the intravenous (i.v.) injections used by previous studies—in order to achieve a lower effective polyI:C dose. Indeed, no effect of the polyI:C alone could be detected in the behavioral tests used although the same i.v. dose has been shown to elicit extensive behavioral deficits (Meyer et al., 2005). DN-DISC1 altered basal levels of interleukin (IL)-1 beta and IL-5 and polyI:C induced secretion of IL-4 and IL-5 in fetal brains 6 h after injection of polyI:C. The other experiments were done on adult males only. The effect of the different manipulations on adult response to acute stress was measured by testing for corticosterone levels at baseline, after a 1 h restraint stress and following 1 h recovery. Although both polyI:C and DN-DISC1 on their own caused a trend of reduced stress-induced corticosterones and no recovery, only the G × E group had significantly lower corticosterones after stress compared to the control group. DN-DISC1 mice have an increased lateral ventricle volume (Pletnikov et al., 2008). Although polyI:C caused increased lateral ventricle volume in wild-type mice, it unexpectedly normalized lateral ventricle volume in the DN-DISC1 mice.

Combination of CaMK-DN-DISC1 × prenatal polyI:C produced new phenotypes not seen with either variable alone: anxiety, as measured by increased peripheral activity in the open field and by open arm time in the elevated plus maze; depression-like behavior, as measured by increased immobility in the forced swim test; and impaired sociability in the three-chamber social test. G × E had no effect on cognitive tests: spontaneous alternation in the Y-maze, object recognition, Morris water maze, PPI.

Finally the authors exploited the Tet-off inducibility of this *CaMK-DN-DISC1* transgene to evaluate the effect of expression timing. In one group, expression of the transgene was induced until weaning (postnatal day 21) and in a second group, expression was induced from weaning onward. The G × E mice behaved normally, implying that continuous expression of the DN transgene is necessary for the G × E effect.

#### CaMK-DN-DISC1 × neonatal polyI:C (Ibi et al., 2010; Nagai et al., 2011)

The CaMK-DN-DISC1 mouse model is very similar to the one employed in the prenatal polyI:C study. The difference is that it expresses the transgene in a constitutive manner. The CaMK-DN-DISC1 genetic model on its own has enlarged lateral ventricles, reduced parvalbumin immunoreactivity, dopaminergic abnormalities and relatively mild behavioral deficits (Hikida et al., 2007; Jaaro-Peled et al., 2013). CaMK-DN-DISC1 mice were injected with polyI:C daily from postnatal day 2–6, which corresponds to the second trimester of human brain development (http://translatingtime.net). This paradigm of neonatal polyI:C injection on its own elicits behavioral abnormalities including impairments in PPI, novel object recognition and social behavior in mice tested at 10–12 weeks of age (Ibi et al., 2009). The DISC1 mutant mice (both males and females) were analyzed starting at 8 weeks of age, or young adulthood. In contrast to the previous study (Ibi et al., 2009), polyI:C on its own did not impair performance in the PPI or novel object recognition paradigms, perhaps due to the age difference. Only G × E resulted in significant reduction in parvalbumin-positive interneurons of the medial prefrontal cortex, reduction in spontaneous alternation in the Y-maze as well as in preference for a novel object in the novel object recognition test (Ibi et al., 2010). The other behavioral tests, sensitivity to MK801, fear conditioning and social interaction were not performed on the G or E groups alone, making it difficult to evaluate any potential G × E interaction. In a follow up paper, the same group tested whether antipsychotics could normalize the behavioral deficits in the CaMK-DN-DISC1 × neonatal polyI:C model. The atypical antipsychotic clozapine normalized the above-mentioned preference in the novel object recognition test, while the typical antipsychotic haloperidol did not (Nagai et al., 2011).

#### PrP-DN-DISC1 × adolescent social isolation (Niwa et al., 2013)

Niwa et al. (2013) is the first publication to introduce this DISC1 mouse model in which the C'-truncated DN-DISC1 is expressed under the control of the widely expressed prion protein promoter. Mice are typically weaned at 3 weeks of age and then housed 5/cage with same-sex littermates. Pre-weaning isolation is extremely stressful and complicated by the physical survival of the pups still dependent on nursing. PrP-DN-DISC1 mice were subjected to a new, milder social stress paradigm of 3 weeks of isolation from 5–8 weeks of age after which they were tested. This timing corresponds to middle and late adolescence, a sensitive period and may therefore model separation from family or general social isolation in adolescent humans. The combination of the DN-DISC1 gene (G) and mild social isolation (E) led to multiple behavioral, cellular and functional deficits whereas neither the environmental nor genetic factors produced deficits on their own. Both males and females were tested and showed similar phenotypes.

PrP-DN-DISC1 mice with 3-week isolation (G × E) displayed multiple behavioral deficits, including decreased prepulse inhibition, increased immobility in a forced swim test, and an augmented locomotion response to methamphetamine challenge when compared to the control, G, and E groups. These abnormal phenotypes resulted from synergistic gene-environment interaction at the cellular level, rather than any gross anatomical or histological mechanism. Body weight and lateral ventricle volume were similar to controls in the G, E, and G × E groups; Nissl staining and Glial fibrillary acidic protein immunostaining also failed to reveal any significant differences. However, the G × E mice displayed significant dopaminergic alterations at the cellular level. Tyrosine hydroxylase expression and total tissue dopamine levels were significantly decreased compared to control, G, and E groups, as was basal extracellular dopamine in the frontal cortex. Consistent with their abnormal locomotor behavior, methamphetamine challenge led to significantly higher dopamine release in the nucleus accumbens of the G × E mice compared to the control, G, and E groups.

Notably, treatment with the glucocorticoid receptor antagonist RU38486 normalized all behavioral and dopaminergic cellular abnormalities in the G × E group. The authors therefore hypothesized that an abnormally high stress-induced corticosterone response in the G × E mice might underlie their dopaminergic pathology. Glucocorticoids mediated projection-specific epigenetic changes in the dopaminergic midbrain of the G × E mice. Specifically, mesocortical dopaminergic projection neurons in the ventral tegmental area exhibited robust DNA methylation of the tyrosine hydroxylase gene promoter, while the mesolimbic projection was unaffected. Furthermore, these DNA methylation levels were maintained in the G × E mice until 20 weeks of age after the transient adolescent isolation. RU38486 normalized the increase in DNA methylation in G × E mice.

Only G × E produced neuronal and behavioral abnormalities in the PrP-DN-DISC1 model; neither the DISC1 mutation nor isolation stress produced any observed pathology on their own. As such, the PrP-DN-DISC1 mice are a promising genetic model for exploring synergistic gene-environment interactions underlying the mechanisms of psychiatric pathology.

#### L100P and Q31L × prenatal polyI:C (Lipina, 2013)

In addition to dominant-negative models, DISC1 point mutants have also been combined with environmental risk factors. These models, identified in a screen of ENU-mutagenized mice, express missense mutations in *Disc1*. The Q31L mutant mice showed depressive-like behavior (deficits in the forced swim test and other measures that were reversed by the antidepressant) whereas L100P mutants exhibited schizophrenia-like behavior (PPI and latent inhibition deficits which were reversed by antipsychotic treatment) (Clapcote et al., 2007). A subsequent study from another group did not reproduce most of these baseline behavioral abnormalities (Shoji et al., 2012) suggesting that the effect of the *Disc1* point mutations is not very robust and may depend on the experimental conditions i.e., subtle environmental factors. The two G × E studies described here used heterozygous *Disc1* point mutants. Heterozygotes exhibit relatively mild phenotypic impairments compared to homozygotes (Clapcote et al., 2007), and as such provide more room for exploring the synergistic interplay between genetic and environmental risk factors. PolyI:C 5 mg/kg i.v. at E9 has been routinely used in environment-only studies and shown to elicit robust phenotypes (Meyer et al., 2005). In this study the authors started with a dose of 5 mg/kg, but pregnant L100P heterozygotes administered polyI:C at 5mg/kg bore no offspring, dead or alive; as a result, they had to decrease the dose to 2.5 mg/kg in order to obtain offspring for behavioral analysis. Litter/offspring size in polyI:C-treated wild type and Q31L mice were unaffected. Thus, they studied Q31L mice with 5 mg/kg polyI:C and L100P mice with 2.5 mg/kg polyI:C. In the assays used, 2.5 mg/kg on its own produced a PPI deficit and 5 mg/kg on its own affected also locomotion in the open field and latent inhibition (LI). Even lower polyI:C dose of 1 mg/kg i.v. may be recommended for G × E studies (Giovanoli et al., 2013).

Since prenatal infection is one of the major epidemiological risk factors for schizophrenia, but not for depression, Lipina et al. (2013) hypothesized that G × E interactions between maternal immune activation produced by prenatal polyI:C would be significantly greater in the L100P mice than the Q31L mice. Heterozygous females were given a single injection of polyI:C on gestational day 9. The Q31L/polyI:C mice exhibited G × E interaction only in risk assessment behavior, meaning that the G × E spent more time scanning the EPM for potential threat than the G or E conditions alone. However, the L100P × polyI:C offspring exhibited a significant exacerbation of schizophrenia-related endophenotypes compared to L100P controls. Deficits in PPI, LI, object recognition, and sociability all demonstrated significant prenatal polyI:C × genotype interactions. Specifically, the L100P × polyI:C group showed a deficit in social motivation but not social recognition—correspondingly, their novel object recognition was intact while spatial object recognition was impaired.

IL-6, a cytokine critical for mediating the effects of prenatal polyI:C, was elevated in the fetal brains of all mice treated with polyI:C; the L100P group, however, exhibited a steeper dose-dependent increase compared to the WT and Q31L groups. It appears that at least PPI and LI behavioral deficits in the L100P mice were causally dependent on prenatal polyI:C, as coadministration of an antibody against IL-6 resulted in normal PPI and LI behavior.

#### L100P and Q31L × social defeat (Haque et al., 2012)

Haque et al. also used L100P or Q31L heterozygous mice for low baseline impairments to enable detection of G × E effect. The mice were subjected to chronic social defeat (CSD) as an environmental stressor. CSD on its own has been shown to induce a depressive/anxiety-like phenotype including hypoactivity in the open field, social avoidance, reduced sucrose consumption, and increased immobility time in the forced swim test (Kudryavtseva et al., 1991; Venzala et al., 2012). This makes it difficult to detect G × E effects in these behavioral tests.

CSD resulted in similar depressive phenotypes for all three genetic groups. L100P and Q31L mice did not display significant abnormality in either forced-swim test immobility or sucrose consumption similar to wild-type controls in both the naïve and defeated conditions. Anxiety-related endophenotypes, on the other hand, revealed significant differences between the two mutant lines and controls, as well as between the mutant lines themselves. L100P and Q31L mice spent less time exploring the open arm of the elevated plus maze than did controls. However, Q31L mice did not display any difference in exploration time in response to CSD, whereas CSD significantly decreased exploration time for wild-type and L100P groups. The L100P defeated mice displayed decreased vertical activity in the open field test compared to all G, E, and control groups. A CSD-related decrease in L100P horizontal activity was also observed during the first 5 min of the open field test, but the difference normalized in subsequent time bins. CSD diminished sociability and preference for social novelty in wild-type and L100P mice. Q31L were a social even without CSD, so no further effect could be detected by addition of CSD stress. CSD did not interact with cognitive deficits in the mutant lines. L100P mice displayed a deficit in PPI, and both L100P and Q31L mice displayed disruptions in LI, but CSD did not exacerbate either endophenotype for any group.

## Discussion

A genetic model with too robust a phenotype may result in a “ceiling effect,” whereby the effects of any potential G × E interactions are occluded. It is therefore more practical to use genetic models with relatively weak phenotypes, such as heterozygotes instead of homozygotes (Ibi et al., 2010; Haque et al., 2012; Lipina et al., 2013; Niwa et al., 2013), or to at least focus on specific assays which are not affected by the G condition on its own (Abazyan et al., 2010). The same is true for the choice of the environmental stressor—to detect G × E effects it is useful to choose a regime which is sub-optimal on its own, such as changing the administration route (Abazyan et al., 2010) or reducing the dose (Lipina et al., 2013) of polyI:C, or developing a new, milder form of social stress paradigm (Niwa et al., 2013).

Interestingly, CaMK-DN-DISC1 × *prenatal* polyI:C synergistically induced anxiety and depression-like phenotypes, but cognitive function was not affected (Abazyan et al., 2010). In contrast, CaMK-DN-DISC1 × *neonatal* polyI:C significantly impacted on cognitive tasks (Ibi et al., 2010). Since these two DISC1 models are very similar, such results imply that specific timing of the polyI:C intervention determines which type of behavioral abnormalities emerge. To test this hypothesis, it would be useful to test a single *DISC1* model against several polyI:C injections at different time points, with subsequent behavioral and histological analyses under identical experimental conditions.

Which biological changes underlie the behavioral abnormalities manifested exclusively as a result of G × E? Ibi et al. found decreased parvalbumin immunoreactivity in the prefrontal cortex but not the hippocampus, hinting at a specific fast-spiking interneuron deficiency (Ibi et al., 2010). Abazyan et al. observed decreased spine density in the dentate gyrus. Their most surprising result was that combining polyI:C with DISC1 suppressed the ventricular enlargement phenotype displayed by either the G or E factor alone (Abazyan et al., 2010). Abazyan et al. and Niwa et al. both looked at monoamines and plasma corticosterone response to environmental stress. In the CaMK-DN-DISC1 × neonatal polyI:C model, no changes were detected in dopamine content or turnover in the frontal cortex or hippocampus, while hippocampal serotonin content was increased (Abazyan et al., 2010). Stress-induced corticosterone levels were attenuated in the G × E condition more than in G or E alone. In the PrP-DN-DISC1 × isolation stress model, frontal cortex and caudate-putamen serotonin were not altered while several dopamine-related measures were lower in the frontal cortex but not in the nucleus accumbens (Niwa et al., 2013). These dopaminergic and behavioral phenotypes are at least in part affected by epigenetic silencing of the tyrosine hydroxylase promoter via the elevated glucocorticoids which were detected only in G × E. The isolation stress on its own was mild enough not to affect plasma corticosterones. Abazyan et al. and Lipina et al. detected effect of G × E on cytokines, but each tested for different cytokines (Abazyan et al., 2010; Lipina et al., 2013). Lipina et al. used anti-IL-6 to rescue polyI:C induced PPI and LI abnormalities in L100P mice, confirming the importance of IL-6 as a mediator of the detrimental effects of maternal immune activation.

Can G × E-induced abnormalities be reversed by pharmacological interventions regularly used in psychiatry? Nagai et al. focusing on schizophrenia, tested antipsychotics against the CaMK-DN-DISC1 × neonatal polyI:C model. Increased sensitivity to MK801, an NMDA receptor antagonist, was rescued by both haloperidol and clozapine. Novel object recognition deficit was normalized only by clozapine treatment, but the sociability deficit could not be rescued by either (Nagai et al., 2011). This model may be useful in exploratory screening for new drugs which are especially effective against social deficits. Based on the increased plasma corticosterone levels in the PrP-DN-DISC1 × social stress model, Niwa et al. treated the mice with the glucocorticoid receptor antagonist RU38486 which normalized all behavioral and dopaminergic cellular abnormalities in the G × E group, most likely via normalization of the tyrosine hydroxylase methylation. RU38486 is uniquely beneficial in psychotic depression, major depression with psychotic features (Flores et al., 2006), suggesting that the PrP-DN-DISC1 × social isolation model may be useful in finding better treatments for this disorder. The above drug treatment trials add predictive validity to their respective animal models and suggest that even abnormalities of neurodevelopmental origin may be reversed with appropriate treatment.

As discussed above, timing is critical. Abazyan et al. induced expression of the CaMK-DN-DISC1 transgene either until weaning or starting at weaning. Interestingly, neither group exhibited the depressive-like behavioral deficits detected with constitutive CaMK-DN-DISC1 expression. Elimination of the behavioral phenotypes after turning off expression during the late postnatal period is encouraging; it implies that these phenotypes are reversible despite being neurodevelopmental in origin (Abazyan et al., 2010). Niwa et al. asked whether adolescent stress-induced epigenetic changes in the tyrosine hydroxylase promoter persist into adulthood. They isolated mice from 5 to 8 weeks but then returned them to group housing until 20 weeks of age. The increase in tyrosine hydroxylase promoter methylation was maintained after 12 weeks of group housing supporting the idea that stress in adolescence can impact the brain for life (Niwa et al., 2013). Demonstrating the importance of the timing in which the mice are tested for potential effects of G × E, the detrimental effect of polyI:C on PPI was not detected at the end of adolescence (8 weeks) but only later in adulthood (16 weeks) (Lipina et al., 2013).

Sex differences are an important and under-researched facet of psychiatric disorders. Schizophrenia starts earlier and is generally more severe in men. The risk for depression is doubled in women compared to men. In depression there is human and animal data on differential effect of early life stress on females (Goel and Bale, 2009). In the CaMK-DN-DISC1 × prenatal polyI:C study (Abazyan et al., 2010), only males were tested, although the same group has shown sex-specific phenotypes in the genetic model alone (Ayhan et al., 2011). Ibi et al. tested both sexes together for CaMK-DN-DISC1 × postnatal polyI:C and did not mention anything about potential sex differences (Ibi et al., 2010). PrP-DN-DISC1 × social isolation was performed on both sexes separately and the results were notably similar for both, suggesting that this paradigm may not be useful to look at sex differences (Niwa et al., 2013). Chronic social defeat is designed to utilize aggressive interactions between males; therefore, only males were tested (Haque et al., 2012).

The recent study combining *Disc1* point mutants with PolyI:C at E9 (Lipina et al., 2013) provides an opportunity to compare the effects of a similar environmental factor on different Disc1 models based on data obtained by the same lab. They found that maternal immune activation interacts with the L100P mutation that causes abnormalities related to schizophrenia while there was no interaction with the Q31L mutation that causes abnormalities related to depression. These results correspond nicely with the epidemiological studies pointing to prenatal infection as a risk factor for schizophrenia but not for depression. The two studies using *Disc1* point mutants also enable us to start to compare the effects of different environmental risk factors on the same model based on data obtained by the same lab. Interestingly, prenatal polyI:C on its own had no effect on sociability, but in combination with L100P it eliminated preference for the stranger (Lipina et al., 2013). On the other hand, CSD reduced sociability both in WT and in L100P mice (Haque et al., 2012). So social impairment can either result from CSD, which is highly relevant for social behavior, or from *Disc1* L100P × prenatal polyI:C interaction—two factors which have no individual effect on sociability.

## Future directions

Exploring a matrix of all risk genes for schizophrenia and their interactions with all environmental risk factors would be exceptionally difficult. Scientists can instead focus on major known susceptibility genes and combine them with environmental factors thought to increase risk, based on epidemiological studies. In order to rigorously dissect the complexity of G × E interactions, both factors must be manipulated independently and the results of G × E must be compared to G and E alone under identical experimental conditions. Ideally, the same genetic model can be exposed to different environmental factors at different times and tested for histological, neurochemical, and behavioral phenotypes without *a priori* assignment of a specific disease model and with equal value assigned to negative results. Such open-ended analysis, although laborious, could suggest how one risk gene can catalyze the emergence of a variety of psychiatric disorders depending on which environmental factor it interacts with (Harvey and Boksa, 2012). DISC1 is an especially attractive candidate for such an approach since it is a risk factor for many different mental disorders. Looking at genetic interactions with an array of environments will still be a simplified model, since additional G × G or E × E interactions probably determine exact outcomes in humans. To further refine our understanding of the *DISC1* × E interaction paradigm, there is a need for greater emphasis on sex differences in mouse models, as well as examination of environmental risk factors such as Toxoplasma gondii exposure, adolescent cannabis abuse and advanced paternal age. In the future, the development of comprehensive “environment interaction profiles” for major risk genes like DISC1 would represent a significant advancement toward the scientific understanding of mental disease pathogenesis.

### Conflict of interest statement

The authors declare that the research was conducted in the absence of any commercial or financial relationships that could be construed as a potential conflict of interest.
